# Krebs von den Lungen-6 (KL-6) Levels in Post-COVID Follow-Up: Differences According to the Severity of COVID-19

**DOI:** 10.3390/jcm12196299

**Published:** 2023-09-29

**Authors:** Carlos Carpio, Ana Qasem, Antonio Buño, Alberto M. Borobia, Francisco Arnalich, Vega Rey, Teresa Lázaro, Pablo Mariscal, Daniel Laorden, Giorgina Salgueiro, Alberto Moreno, Concepción Peiró, Óscar Lorenzo, Rodolfo Álvarez-Sala, on behalf of the COVID@HULP Working Group, POSTCOVID@HULP Working Group

**Affiliations:** 1 Pneumology Department, Hospital Universitario La Paz, Instituto de Investigación Hospital Universitario La Paz (IdiPAZ), Universidad Autónoma de Madrid, 28046 Madrid, Spain; teresa.lazaro@salud.madrid.org (T.L.); pablo.mariscal@salud.madrid.org (P.M.); laordendaniel@gmail.com (D.L.); rodolfo.alvarezsala@salud.madrid.org (R.Á.-S.); 2 Clinical Analytics Department, Hospital Universitario La Paz, IdiPAZ, Universidad Autónoma de Madrid, 28049 Madrid, Spain; antonio.buno@salud.madrid.org; 3 Clinical Pharmacology Department, Hospital Universitario La Paz, IdiPAZ, Universidad Autónoma de Madrid, 28049 Madrid, Spain; a.borobia@gmail.com (A.M.B.); verema00@gmail.com (V.R.); 4 Internal Medicine Department, Hospital Universitario La Paz, IdiPAZ, Universidad Autónoma de Madrid, 28049 Madrid, Spain; francisco.arnalich@gmail.com (F.A.); giorgisalgueiro@hotmail.com (G.S.); a.moreno@salud.madrid.org (A.M.); 5 Pharmacology Department. Universidad Autónoma de Madrid, IdiPAZ, 28049 Madrid, Spain; concha.peiro@uam.es; 6 Laboratory of Diabetes and Vascular pathology, IIS, Fundación Jiménez Díaz, CIBERDEM, Universidad Autónoma de Madrid, 28049 Madrid, Spain; olorenzo@fjd.es

**Keywords:** post-acute COVID-19 syndrome, KL-6 antigen, human, COVID-19

## Abstract

To evaluate KL-6 levels in medium-term post-COVID and to compare them in three groups categorised by the severity of COVID-19, we conducted a real-world, retrospective, cohort study. Data from the COVID-19 episode and follow-up during the post-COVID phase were extracted from the COVID@HULP and POSTCOVID@HULP databases, respectively. For the post-COVID period we included demographics, medical history, symptoms, quality of life, physical activity, anxiety and depression status and laboratory results. Patients were categorised into three groups based on the severity of COVID-19: Group 1 (inpatient critical), Group 2 (inpatient non-critical) and Group 3 (hospitalised at home). KL-6 was measured during the follow-up of the three groups. In all, 802 patients were included (Group 1 = 59; Group 2 = 296; Group 3 = 447 patients). The median age was 59 years (48–70), and 362 (45.2%) were males. At admission, fibrinogen and ferritin levels were lower in Group 3 than in the other groups (*p* < 0.001). Follow-up data were obtained 124 days (97–149) after the diagnosis of COVID-19. The median levels of fibrinogen, ferritin and KL-6 at follow-up were 336 mg/dL (276–413), 80.5 ng/mL (36–174.3) and 326 U/mL (240.3–440.3), respectively. KL-6 levels were lower in Group 3 than in the other groups (298 U/mL (231.5–398) vs. 381.5 U/mL (304–511.8) (Group 1) and 372 U/mL (249–483) (Group 2) (*p* < 0.001)). KL-6 was associated with ferritin (*p* < 0.001), fibrinogen (*p* < 0.001), D-dimer (*p* < 0.001) and gamma-glutamyl transferase (*p* < 0.001). KL-6 levels are less elevated at medium-term post-COVID follow-up in patients with mild COVID-19 than in those with moderate or severe disease. KL-6 is associated with systemic inflammatory, hepatic enzyme and thrombosis biomarkers.

## 1. Introduction

Although COVID-19 mainly affects the lungs, during the course of the disease, it can trigger multi-systemic infection and impact heart and kidney function, leading to the appearance of coagulopathy [1,2,3,4,5]. Thus, COVID-19 has a spectrum of infection severity ranging from mild to critical disease [6]. In this context, a poor prognosis with need for admission to intensive care units is more likely in individuals with abnormalities in inflammatory parameters than in those without abnormalities, to the extent that the latter could be managed as outpatients [7,8]. The persistence of post-COVID-19 symptoms, known as long COVID, can be found in more than 50% of patients during follow-up and is more frequent in patients admitted to intensive care units than in those treated in hospital wards [9,10]. Various hypotheses have been proposed for the pathophysiology of long COVID, including one related to a scenario of immunologic aberrations and inflammatory damage (including neutrophil and monocyte invasion, cytokine expression and increased levels of fibrinogen and C-reactive protein (CRP)) [11,12]. Zhou et al. [13] demonstrated that levels of inflammatory cytokines remain higher in severely affected individuals in comparison with mildly affected or healthy controls 3 months after the acute COVID-19 episode and that these changes were associated with functional or radiological respiratory abnormalities.

Serum Krebs von den Lungen-6 (KL-6) is a high-molecular-weight mucin-like glycoprotein produced by type II pneumocytes and bronchial cells [14]. It is elevated in interstitial lung diseases and acute respiratory distress syndrome and reflects alveolar epithelial injury [14,15]. In COVID-19, serum KL-6 levels combined with inflammatory parameters such as CRP act as biomarkers of poor prognosis and are higher in severe disease [16,17,18]. These levels can also predict the appearance of pulmonary fibrotic sequelae, both in patients treated in intensive care units and in hospital wards [19]. To date, however, studies evaluating the KL-6 pattern in long COVID are scarce, composed of small cohorts or individuals and only reporting short-term follow-up. The study by Deng et al. [20], comprising a cohort of 166 patients with COVID-19, 17 of them severe, observed that KL-6 levels 100 days after COVID-19 onset were related to KL-6 levels within 10 days of diagnosis and acted as a potent predictor for lung injury prognosis. In our study, we aimed to consider the variance of KL-6 levels at medium term in three different populations diagnosed with COVID-19: severe, non-severe treated at hospital and non-severe treated at home.

## 2. Materials and Methods

### 2.1. Study Design and Objectives

This was a real-world, retrospective, single-centre, cohort study. Our primary objective was to evaluate differences in KL-6 levels during the medium-term follow-up of patients admitted with COVID-19. As a secondary objective, we also analysed associations between KL-6 and systemic serum biomarkers.

### 2.2. Patient Population and COVID-19 Database

We included all individuals 18 years or older with a diagnosis of COVID-19 in outpatient follow-up 3 to 6 months after admission to a 1286-bed hospital in Madrid (La Paz University Hospital). All the patients were treated during follow-up in the specialised Post-COVID Unit from July 2020 to January 2021, and in-person medical visits were required for follow-up.

At our institution, all data on individuals admitted with a COVID-19 diagnosis have been collected in a database, previously described in the literature [7,21], including 3934 patients consecutively treated in the hospital’s Emergency Department. This database, called COVID@HULP, includes 372 variables. Follow-up information was registered in a separate database called POSTCOVID@HULP, which includes 105 variables grouped into demographics, medical history, symptoms, vital signs, quality of life, physical activity, laboratory results and anxiety and depression status (extracted from different hospital data management systems). For the purposes of our study, we extracted data on age, sex, smoking status, comorbidities and oxygen therapy requirements during admission to hospital. We included all the information recorded in the POSTCOVID@HULP database regarding follow-up.

Patients were categorised into 3 groups based on COVID-19 severity:

- Group 1: Severe COVID-19. Critical inpatient group. Patients requiring admission to the intensive and/or intermediate care units.
- Group 2: Moderate COVID-19. Non-critical inpatient group. Patients who were hospitalised but who did not require admission to the intensive and/or intermediate care units.
- Group 3: Mild COVID-19. Hospital-at-home care group. Treated at home.

This study was approved by the Research Ethics Committee of the Hospital Universitario La Paz (Approval code: PI-4234, Date of approval: 6 July 2020). Written informed consent was obtained from all patients for the follow-up.

### 2.3. Variables

KL-6 was measured using Lumipulse^®^ G KL-6 (Krebs von den Lungen, Tokyo, Japan) immunoreaction cartridges designed for in vitro diagnostic use with the LUMIPULSE G System (Fujirebio, Tokyo, Japan). The assay utilises proven CLEIA (ChemiLuminescent Enzyme Immunoassay)(Tokyo, Japan) technology.

Quality of life: For this measure, we applied the Euro Quality of Life (EuroQoL) questionnaire, a standardised instrument for measuring health-related quality of life, assessed by severity level and dimension with a more general visual analogue scale (VAS) assessment. A third element of this questionnaire is the index of social values for each health state generated by VAS and time trade-off (TTO) scores. The descriptive system comprises 5 dimensions of health (mobility, self-care, usual activities, pain/discomfort and anxiety/depression), each of which has 3 response levels of severity (no problems, some problems and extreme problems) [22].

Daily physical activity: The London Chest Activity of Daily Living (LCADL) scale questionnaire was used to describe this variable. It comprises 15 items that measure the degree of perceived dyspnoea during activities of daily living divided into 4 components or domains (self-care and physical, leisure and domestic activities). The sum of the scores (in a theoretical range of 0 to 75) determines the degree of impairment. A higher score indicates an increased perception of dyspnoea while performing activities of daily living [23].

Emotional state: To evaluate anxiety and depression, we applied the Hospital Anxiety and Depression Scale (HADS) questionnaire, comprising 14 questions divided into 2 subscales: anxiety (7 items) and depression (7 items), with scores ranging from 0 to 21. The total score (anxiety and depression) ranges from 0 to 42 on a 4-point Likert scale, with an interval ranging from 0 to 3, in which 0 is “never” and 3 is “virtually all day” [24].

### 2.4. Statistical Analysis

The quantitative variables were expressed as medians with interquartile range (IQR). For the categorical variables, frequencies and proportions were employed. Prior to the analyses, a normality analysis was performed with the Shapiro–Wilk test; Student’s *t*-test was used for the parametric analysis and the Mann–Whitney U test for non-parametric analyses. Spearman’s correlation was applied for correlations between quantitative variables. For associations between qualitative variables, we used the chi-squared test (or Fisher’s test when necessary). Given that our study included 3 groups of patients, a Bonferroni adjustment was made to prevent the accumulation of error. As a result, statistical significance was set at *p* ≤ 0.016. Statistical analyses were performed with R version 4.0.4.

## 3. Results

### 3.1. Baseline Characteristics at Admission

A total of 802 patients were included (Group 1 = 59, Group 2 = 296, Group 3 = 447). The median age was 59 (48–70) years, and 362 (45.2%) were male. The main comorbidities were systemic hypertension (36.4%), dyslipidaemia (30.4%) and diabetes mellitus (15.6%). Notable laboratory results at admission included absolute lymphocyte count 1.5 × 103/µL (0.9–2), fibrinogen 408.5 mg/dL (300–658), ferritin 164 ng/mL (62–440) and D-dimer 532 ng/mL (322–1113) (Table 1).

### 3.2. Baseline Characteristics Compared by Group

The patients in Group 3 were younger (*p* < 0.001) and had a lower proportion of men (*p* < 0.001) and current smokers (*p* = 0.001) than the other two groups. The prevalence of systemic hypertension (*p* < 0.001) and dyslipidaemia (*p* < 0.001) was also lower in Group 3 than in Groups 1 and 2. Oxygen therapy was required in 7.1% of the patients in Group 3 over the course of the disease, whereas an oxygen supply was required in 96.4% and 88% of Group 1 and 2 patients, respectively. Systemic inflammatory biomarkers such as CRP (*p* < 0.001), fibrinogen (*p* < 0.001) and ferritin (*p* < 0.001) were lower in Group 3 than in the other groups (Table 1).

### 3.3. Characteristics of Each Group at Follow-Up

Follow-up was performed 124 (97–149) days after the COVID-19 diagnosis. Dyspnoea (66.6%) and fatigue (44.5%) were the most reported symptoms. In terms of quality of life, the TTO and VAS values were 0.8 (0.7–0.9) and 0.7 (0.6–0.8), respectively. With respect to systemic inflammatory biomarkers, elevated CRP levels were observed in 58.5% of patients, and the median levels of fibrinogen and ferritin were 336 (276–413) mg/dL and 80.5 (36–174.3) ng/mL, respectively. The median KL-6 levels were 326 U/mL (240.3–440.3) (Table 2).

When the groups were compared, dyspnoea (*p* = 0.044), fatigue (*p* = 0.01) and myalgia (*p* = 0.047) were more prevalent in Group 3 than in Group 2, whereas the EuroQoL, TTO (*p* = 0.034) and VAS (*p* = 0.031) values were higher in Group 2 than in Group 1. CRP levels of > 0.5 mg/L were more frequently observed in Group 1 than in the other groups. The KL-6 levels were lower in Group 3 than in the other groups; however, no significant differences were observed among the groups in relation to ferritin and fibrinogen at follow-up (Table 2).

### 3.4. Associations with KL-6

KL-6 was positively associated with white blood cell count (rho = 0.099; *p* = 0.004), absolute lymphocyte count (rho = 0.099; *p* = 0.044) and serum levels of fibrinogen (rho = 0.197; *p* < 0.011), ferritin (rho = 0.204; *p* < 0.001), D-dimer (rho = 0.199; *p* < 0.001), gamma-glutamyl transferase (rho = 0.176; *p* < 0.001), alanine aminotransferase (rho = 0.120; *p* = 0.016) and aspartate aminotransferase (rho = 0.124; *p* = 0.014) (Table 3).

## 4. Discussion

Our results demonstrate that serum KL-6 in medium-term follow-up is lower in patients hospitalised at home than in those treated in hospital wards or requiring admission to an intensive and/or intermediate care unit. In addition, we found that serum KL-6 correlated with inflammatory, hepatic enzyme and thrombosis biomarkers.

Other studies have reported elevated KL-6 levels at admission and during hospitalisation in patients diagnosed with COVID-19 compared with healthy controls [25]. Moreover, serum levels of this glycoprotein have been found to be higher in severe than in non-severe disease [26,27], independent of the parameters used to describe COVID-19 severity (respiratory rate, resting SpO_2_, ratio of partial pressure of oxygen to fraction of inspired oxygen, need for supplemental oxygen, extension of lung infiltrates, multiple organ dysfunction). Evidence of this elevation in a short-term follow-up has been described by Deng et al. [20] in a reduced population (166 patients, 17 with severe COVID-19). These authors also found that serum KL-6 at follow-up (100 days post-COVID-19 onset) correlated with KL-6 levels within 10 days post-onset. Unlike our study, however, they did not compare serum KL-6 in the two groups included (mild and severe/critical COVID-19) during follow-up. In our cohort, we found that patients with COVID-19 treated at home (mild COVID-19) presented a lower KL-6 increase compared with hospitalised patients (both in critical units and inpatient wards). Furthermore, even at medium-term follow-up, our results indicate that serum KL-6 remains elevated compared with levels described in healthy controls [28] and that, remarkably, this also occurs in patients who do not require hospitalisation (mild COVID-19).

KL-6, a sub-molecule of the glycoprotein mucin 1 (MUC1) expressed in type II pneumocytes and respiratory bronchiolar epithelial cells [29,30], plays an essential role in the pathophysiological processes of respiratory diseases; it is increased in interstitial lung diseases (most notably in the evaluation of idiopathic pulmonary fibrosis) and acute respiratory distress syndrome [14,15]. In interstitial lung diseases, it identifies patients with a higher risk for mortality, and its changes over a 1-year period act as a predictor for disease progression [31,32]. Given that patients with COVID-19 can present bilateral and multifocal lung lesions in computed tomography scans [25], KL-6 has been proposed as a biomarker of alveolar damage in this disease. Results from the study by Awano et al. [27] have demonstrated that patients with severe COVID-19 have higher serum KL-6 compared with those with non-severe disease, both at diagnosis and 1 week afterwards. Subsequently, Bergantini et al. [16] found that, in addition to higher KL-6 levels detected in patients with severe disease, its concentrations correlated with systemic biomarkers such as interleukin-6 and CRP. These studies and five others were included in the meta-analyses by Naderi and Rahimzadeh [17], which confirmed the presence of higher serum KL-6 in patients with severe COVID-19 compared with mild-to-moderate COVID-19, as well as with healthy controls. Possible explanations for these findings focus on the severe alveolar epithelial damage caused by viral replication and its repercussion on KL-6 secretion, which in the context of overproduction of fibronectin, thrombin and epidermal growth factor could contribute to the development of structural damage to the lung [17,33]. Diffuse alveolar damage, confirmed in pulmonary pathology studies [34,35], directly impacts the oxygen levels of patients diagnosed with COVID-19. Thus, the ratio of the arterial partial pressure of oxygen to the fraction of inspired oxygen on admission, a key component of alveolar damage and acute respiratory distress syndrome, is correlated with serum KL-6 levels in COVID-19 [26,33,36].

Our findings provide evidence of a correlation between serum KL-6 and biomarkers of coagulation dysfunction (D-dimer, fibrinogen) at follow-up. Karadeniz et al. [25] described similar associations upon admission of patients with COVID-19 in a reduced population (59 patients). The longitudinal retrospective cohort study by Deng et al. [20] reported that KL-6 correlated with D-dimer and fibrinogen, particularly in severe COVID-19. However, these authors did not specify whether these associations were evaluated at admission, during follow-up (100 days post-COVID-19 onset) or over the entire study period. Our results demonstrate, on the one hand, that coagulation dysfunction persists in long-term follow-up, especially in cases of severe COVID-19 and, on the other, that KL-6 is associated with this dysfunction. All findings regarding our cohort are supported by the microangiopathy and pulmonary thrombosis detected in the histologic analysis of pulmonary vessels in patients with COVID-19 [34,35]. Such outcomes are important, given that higher biomarkers of coagulation dysfunction, such as D-dimer and fibrinogen, indicate a more aggressive disease and therefore allow for the identification of patients with a greater risk of severe disease at the time of admission [37]. In the case of post-COVID, elevated KL-6 levels and their relationship with D-dimer and fibrinogen could be interpreted as a persistence of alveolar damage and microangiopathy insofar as these occur during the acute phase of the disease; however, this hypothesis remains to be proven.

In addition, we observed that inflammatory biomarkers and hepatic transaminases are associated with serum KL-6. Karadeniz et al. [25] found similar results at admission but not at follow-up. There was no difference between our patients and those of their study in serum transaminase values at admission. However, these authors did not detect significant correlations between serum KL-6 and transaminases. In addition, our study found a lower reported prevalence of liver disease as an associated comorbidity at admission (2.1%), whereas Karadeniz et al. [25] did not report any such prevalence in their cohort. Several pathways have been proposed in the pathogenesis of liver injury in patients with COVID-19, including the cytopathic effect of the virus, cytokine storm, systemic inflammatory response syndrome, thrombosis, hypoxia and sepsis [38]. In addition, hepatocytes and cholangiocytes express angiotensin-converting enzyme receptor 2 during long COVID-19, thereby incurring a mild derangement of hepatic biomarkers that sometimes result in the development of a cholangiopathy [39].

Limitations to our study include its retrospective design and the need to address the limitations that such designs imply (control for confounding factors and biases). However, to conduct this study we used the data recorded on platforms (COVID@HULP and POSTCOVID@HULP) specifically designed for the analysis and exploration of data from patients admitted for COVID-19, which allowed us to control some of these disadvantages. Another limitation to our study is that serum KL-6 was not recorded at admission; therefore, we were unable to evaluate the evolution of this biomarker of alveolar damage over time. An ideal study might compare KL-6 clearance curves over a follow-up period in these groups rather than analysing this biomarker only once over time. As a third limitation, it is important to emphasise that Group 3 was composed of patients hospitalised at home and not by outpatients, given that it could generate biases in our conclusions. During the pandemic and due to limited health resources, our institution was forced to create a home hospitalisation unit for younger patients who had no risk factors for severe COVID-19, who at admission did not have respiratory failure or who only required oxygen therapy with very low oxygen flows, who had good family support and whose chest X-ray did not show pneumonia or involvement of a single lobe. This group of patients (corresponding to Group 3) were followed-up daily through phone calls made by hospital physicians who, in the event of any complication, performed an in-person medical evaluation. As shown in Table 1, patients in Group 3 were younger than those in the other two groups, had a lower percentage of comorbidities and only 7% required oxygen therapy at admission. Also, it should be emphasised that we did not included data from radiologic studies or lung function tests. Lastly, the single-centre setting and the relatively low number of patients should be included as limitations of this study.

## 5. Conclusions

In this study, we found that KL-6 levels are elevated in the medium-term follow-up of post-COVID patients; however, this increase is lower in patients with mild COVID-19 than in those with moderate or severe disease. In addition, our findings indicated that KL-6 is associated with systemic inflammatory, hepatic enzyme and thrombosis biomarkers. Lastly, there is a need for additional prospective studies to confirm our results.

## Figures and Tables

**Table 1:** Baseline patient characteristics at admission by place of hospitalisation.

	Total (*n* = 802)	Group 1 (*n* = 59)	Group 2 (*n* = 296)	Group 3 (*n* = 447)	*p* *
Men, n (%)	362 (45.2)	40 (67.8)	162 (54.9)	160 (35.8)	<0.001
Age, years	59 (48–70)	61 (54–68)	68 (57–77)	53 (42–62)	<0.001
Current smoker, n (%)	30 (7)	7 (23.3)	20 (6.9)	3 (2.8)	0.001
Comorbidities					
Obesity, n (%)	63 (14.7)	9 (29)	47 (16.4)	7 (6.4)	0.003
Systemic hypertension, n (%)	159 (36.4)	15 (48.4)	127 (43.2)	17 (15.2)	<0.001
Dyslipidaemia, n (%)	133 (30.4)	11 (35.5)	108 (36.6)	14 (12.5)	<0.001
Diabetes mellitus, n (%)	68 (15.6)	7 (22.6)	52 (17.7)	9 (8)	0.030
COPD, n (%)	40 (9.2)	3 (9.7)	26 (8.9)	1 (0.9)	0.015
Neoplastic disease, n (%)	37 (8.5)	2 (6.5)	33 (11.2)	2 (1.8)	0.009
Kidney disease, n (%)	17 (3.9)	0	17 (5.8)	0	0.014
Liver disease, n (%)	9 (2.1)	1 (3.2)	8 (2.7)	0	0.204
Oxygen therapy at admission	291 (69.5)	27 (96.4)	257 (88)	7 (7.1)	<0.001
Laboratory results at admission					
White blood cell count, ×10^3^/µL	5.9 (4.8–7.6)	8.4 (7.3–11.1)	5.9 (4.7–7.4)	5.8 (5.1–7.4)	<0.001
Absolute lymphocyte count, ×10^3^/µL	1.5 (0.9–2)	1.1 (0.8–1.3)	1.3 (0.8–1.9)	1.9 (1.5–2.3)	<0.001
Platelet count, ×10^3^/µL	257 (200–325)	355 (254.5–446.5)	242.5 (188–315)	265.5 (224.5–321.2)	<0.001
C–reactive protein, mg/L	41.4 (7.6–124.9)	136.3 (43.4–249.1)	78 (21.4–139.9)	1.7 (0.5–7.8)	<0.001
Fibrinogen, mg/dL	408.5 (300–658)	391 (240–658)	489 (325.3–725)	328 (260–391)	<0.001
Ferritin, ng/mL	164 (62–440)	555 (355–1012)	209 (84–579)	66 (28–131)	<0.001
D–dimer, ng/mL	532 (322–1.113)	2368 (841–6084)	600 (368.8–1109.5)	270 (160–402.5)	<0.001
Glomerular filtration rate, mL/min/1.73 m^2^	79 (63.5–86)	68.5 (0.5–0.9)	79 (62.3–85.8)	84 (77–89)	<0.001
Gamma–glutamyl transferase, UI/L	32 (19–69.8)	123 (43.5–303.5)	38 (21–76)	20 (15–26.3)	<0.001
Alanine aminotransferase, UI/L	27 (19–49)	64 (39–102.5)	28 (19.5–50.5)	21 (18–33)	<0.001
Aspartate aminotransferase, UI/L	26.5 (18–41)	52 (25.5–81.5)	28 (20–43)	18 (15–27)	<0.001

Comparisons between groups by unpaired samples using Student’s *t*-test, Mann–Whitney U test and chi-squared test. Group 1: critical inpatient group; Group 2: non-critical inpatient group; Group 3: hospital-at-home group. Abbreviations: COPD = chronic obstructive pulmonary disease. * *p*: compares differences between Groups 1, 2 and 3.

**Table 2:** Baseline characteristics at patient follow-up by place of hospitalisation.

	Total (*n* = 802)	Group 1 (*n* = 59)	Group 2 (*n* = 296)	Group 3 (*n* = 447)	*p* *
Symptomatology					
Dyspnoea, n (%)	351 (66.6)	34 (66.7)	117 (59.1)	200 (71.9)	0.014
Fatigue, n (%)	233 (44.5)	22 (43.1)	69 (35.4)	142 (51.1)	0.003
Myalgia, n (%)	223 (42.4)	26 (51)	67 (34)	130 (46.8)	0.009
Cough, n (%)	106 (20.1)	14 (27.5)	33 (16.7)	59 (21.2)	0.184
Thoracic pain, n (%)	79 (14.8)	7 (13.7)	26 (13.1)	46 (16.3)	0.598
Fever, n (%)	23 (4.4)	0	6 (3)	17 (6.1)	0.073
Vital signs and anthropometry					
Heart rate, beats per minute	83 (74–91)	80 (73.5–83.5)	81 (72–90)	84 (75–93)	0.470
SpO_2_, %	97 (96–98)	97 (96–98)	96 (95–97)	97 (96–98)	<0.001
Body mass index, kg/m^2^	25 (23–29)	27 (25–31)	25 (24–29)	25 (23–29)	0.038
HADS	12 (7–18)	11 (6.5–17)	11 (6–17.3)	12 (7–19)	0.298
EuroQoL					
Time trade-off value	0.8 (0.7–0.9)	0.7 (0.3–0.9)	0.8 (0.7–0.9)	0.8 (0.6–0.9)	0.041
Visual analogue scale value	0.7 (0.6– 0.8)	0.6 (0.4–0.8)	0.7 (0.6–0.8)	0.7 (0.6–0.8)	0.038
EuroQoL-visual analogue scale	70 (55–80)	70 (55–82.5)	70 (60–80)	60 (50–73.8)	0.010
LCADL scale	20 (16–28)	19 (15–30.5)	20 (16–27)	20 (16–28.5)	0.541
LCADL self-care domain	4 (4–6)	5 (4–7)	4 (4–6)	4 (4–6)	0.345
LCADL domestic activities domain	6 (6–11)	6 (4–12)	6 (6–10)	6 (6–12)	0.322
LCADL physical activities domain	4 (3–6)	4 (3–6)	4 (3–6)	4 (3–6)	0.542
LCADL leisure activities domain	4 (3–6)	4 (3–6.5)	4 (3–5)	4 (3–6)	0.485
Laboratory results at 6 months					
White blood cell count, ×10^3^/µL	6.5 (5.4–7.6)	6.5 (5.4–7.9)	6.5 (5.4–7.6)	6.4 (5.4–7.6)	0.908
Absolute lymphocyte count, ×10^3^/µL	1.9 (1.6–2.4)	2.2 (1.6–2.7)	1.9 (1.5–2.7)	1.9 (1.6–2.4)	0.082
Platelet count, ×10^3^/µL	244 (207–291)	258.5 (209.5–313.5)	226 (191–283.5)	251 (215–293)	<0.001
C-reactive protein, mg/L	2.7 (0.6–14.4)	4.9 (1.2–17.6)	2.5 (0.7–10.5)	2.6 (0.5–15.7)	0.003
Fibrinogen, mg/dL	336 (276–413)	343 (302.3–462)	336.5 (282–409)	336 (271–412)	0.320
Ferritin, ng/mL	80.5 (36–174.3)	107 (64–166)	89.5 (42–164.5)	75 (31–183)	0.340
D-dimer, ng/mL	330 (210–565)	450 (230–965)	370 (250–610)	290 (190–485)	<0.001
Glomerular filtration rate, mL/min/1.73 m^2^	78 (67–84)	81 (72–82)	77 (64.8–84)	78 (69–86)	0.491
Gamma-glutamyl transferase, UI/L	23 (16–35)	33.5 (17–43.5)	24 (18–34)	21 (15–33)	0.003
Alanine aminotransferase, UI/L	23 (17–34)	24 (17–40)	23 (18–33)	24 (17–35)	0.929
Aspartate aminotransferase, UI/L	19 (15–26)	19 (14.5–26)	20 (16–27)	19 (15–25)	0.394
KL-6, U/mL	326 (240.3–440.3)	381.5 (304–511.8)	372 (249–483)	298 (231.5–398)	<0.001

Data expressed as median (interquartile range) or number (percentage). Comparisons between groups by unpaired samples using Student’s t-test, Mann–Whitney U test and chi-squared test. Group 1: critical inpatient group; Group 2: non-critical inpatient group; Group 3: hospital-at-home group. Abbreviations: EuroQoL = Euro quality of life questionnaire; HADS = Hospital Anxiety and Depression scale; KL-6= Krebs von den Lungen-6; LCADL = London Chest Activity of Daily Living scale; SpO_2_ = arterial oxygen saturation. * *p*: compares differences between Groups 1, 2 and 3.

**Table 3:** Associations with serum KL-6 levels.

	KL-6 Levels	
	rho	*p*
SpO_2_, %	−0.006	0.986
EuroQoL		
Time trade-off value	0.028	0.565
Visual analogue scale value	0.029	0.547
EuroQoL-VAS	0.107	0.152
LCADL scale	0.016	0.737
LCADL self-care domain	0.073	0.139
LCADL domestic activities domain	−0.032	0.524
LCADL physical activities domain	0.049	0.322
LCADL leisure activities domain	0.037	0.454
Laboratory results at follow-up		
White blood cell count, ×10^3^/uL	0.099	0.004
Absolute lymphocyte count, ×10^3^/µL	0.099	0.044
Platelet count, ×10^3^/µL	−0.41	0.407
Fibrinogen, mg/dL	0.197	<0.001
Ferritin, ng/mL	0.204	<0.001
D-dimer, ng/mL	0.199	<0.001
Glomerular filtration rate, mL/min/1.73 m^2^	−0.038	0.622
Gamma-glutamyl transferase, UI/L	0.176	<0.001
Alanine aminotransferase, UI/L	0.120	0.016
Aspartate aminotransferase, UI/L	0.124	0.014

Associations between variables by Spearman’s correlation coefficient. Abbreviations: EuroQoL = Euro quality of life questionnaire; KL-6 = Krebs von den Lungen-6; LCADL = London Chest Activity of Daily Living scale; SpO_2_ = arterial oxygen saturation.

## Data Availability

The original contributions presented in this study are included in the article. Further enquiries can be directed to the corresponding author.

## Supplementary Materials

The following supporting information can be downloaded at: https://www.mdpi.com/article/10.3390/jcm12196299/s1, Table S1: COVID@HULP working group; Table S2: POSTCOVID@HULP working group.
