# Supplementary material for: Krebs von den Lungen-6 (KL-6) Levels in Post-COVID Follow-Up: Differences According to the Severity of COVID-19
Source: J Clin Med. 2023 Sep 29;12(19):6299. doi: 10.3390/jcm12196299 (PMC10573402; doi:10.3390/jcm12196299)
Supplement: Supplementary file 1 [file jcm-12-06299-s001.zip › jcm-2611527-SI.pdf]

Journal of
*Clinical Medicine*

MDPI

**Table S1.** COVID@HULP Working Group.

| SURNAME | NAME |
|---|---|
| Scientific Committee: | |
| Arribas | José Ramón |
| Borobia | Alberto M. |
| Carcas-Sansuán | Antonio |
| Frías | Jesús |
| Ramírez | Elena |
| Martín-Quirós | Alejandro |
| Quintana-Díaz | Manuel |
| Mingorance | Jesús |
| Arnalich | Francisco |
| Moreno | Francisco |
| Carlos Figueiras | Juan |
| García-Arenzana | Nicolás |
| **Microbiology** | |
| Montero Vega | María Dolores |
| Romero Gómez | María Pilar |
| Toro-Rueda | Carlos |
| García-Bujalance | Silvia |
| Ruiz-Carrascoso | Guillermo |
| Cendejas-Bueno | Emilio |
| Falces-Romero | Iker |
| Lázaro-Perona | Fernando |
| Ruiz-Bastián | Mario |
| Gutiérrez-Arroyo | Almudena |
| Girón De Velasco-Sada | Patricia |
| Dahdouh | Elie |
| Gómez-Arroyo | Bartolomé |
| García-Sánchez | Consuelo |
| Guedez-López | Virginia |
| Bloise-Sánchez | Iván |
| Alguacil-Guillén | Marina |
| Liras-Hernández | Maria Gracia |
| Sánchez-Castellano | Miguel Angel |
| García-Clemente | Paloma |
| González-Donapetry | Patricia |
| San José-Villar | Sol |
| de Pablos Gómez | Manuela |
| Gómez-Gil | Rosa |
| Corcuera-Pindado | Maria Teresa |
| Rico-Nieto | Alicia |
| Pharmacy | |
| Herrero | Alicia |
| Laboratory | |
| Prieto Arribas | Daniel |
| Oliver-Saez | Paloma |
| Mora Corcovado | Roberto |
| Fernández-Calle | Pilar |

| | |
|---|---|
| Alcaide Martín | Mª José |
| Díaz-Garzón Marco | Jorge |
| Fernández-Puntero | Belén |
| Nuñez Cabetas | Rocío |
| Crespo Sánchez | Gema |
| Rodriguez Fraga | Olaia |
| Mendez del Sol | Helena |
| Duque Alcorta | Marta |
| Gomez Rioja | Rubén |
| Sanz de Pedro | María |
| Pascual García | Lydia |
| Segovia Amaro | Marta |
| Iturzaeta Sánchez | Jose Manuel |
| Rodriguez Gutiérrez | Mercedes |
| Perez Garcia Morillon | Amparo |
| Martinez Gallego | Miguel Angel |
| Fabre Estremera | Blanca |
| Martinez | Estefaní |
| Moreno Parra | Isabel |
| Rodriguez Roca | Neila |
| Ortiz Sánchez | Daniel |
| Simon Velasco | Manuela |
| Gabriela Tomoiu | Ileana |
| Pizarro Sanchez | Cristina |
| Montero San Martín | Blanca |
| Qasem Moreno | Ana Laila |
| Gómez López | Marta |
| Casares Guerrero | Ismael |
| Buño Soto | Antonio |
| **Radiology** | |
| Martí de Gracia | Milagros |
| Parra Gordo | Luz |
| Diez Tascón | Aurea |
| Ossaba Vélez | Silvia |
| Pinilla | Inmaculada |
| Cuesta | Emilio |
| Fernández-Velilla | María |
| Torres | Maria Isabel |
| Garzón. | Gonzalo |
| Preventive Medicine | |
| Pérez-Blanco | Verónica |
| Quintás-Viqueira | Almudena |
| San Juan | Isabel |
| Cantero-Escribano | José Miguel |
| Pérez-Romero | César |
| Castro-Martínez | Mercedes |
| Hernández-Rivas | Lucia |
| Pedraz | Teresa |
| Fernández-Bretón | Eva |
| García-Vaz | Claudia |
| Robustillo-Rodela | Ana |

| | |
|---|---|
| Emergency | |
| Torres Santos-Olmo | Rosario María |
| Rivera Núñez | Angélica |
| Fernández Fernández | Ignacio |
| Noguerol Gutiérrez | Marina |
| Martínez Virto | Ana María |
| González Viñolis | Manuel |
| Cabrera Gamero | Regina |
| Mayayo Alvira | Rosa |
| Marín Baselga | Raquel |
| Lo-Iacono García | Victoria |
| Lerín Baratas | Macarena |
| Romero Gallego-Acho | Paloma |
| Reche Martínez | Begoña |
| Tejada Sorados | Renzo |
| Rico Briñas | Mikel |
| Deza Palacios | Ricardo |
| Fabra Cadenas | Sara |
| Arroyo Rico | Isabel |
| Dani Ben-Abdellah | Lubna |
| Labajo Montero | Laura |
| Soriano Arroyo | Rubén |
| López Corcuera | Lorena |
| Calvin García | Elena |
| Martínez Álvarez | Susana |
| López-Tappero Irazábal | Laura |
| Pilares Barco | Martín |
| González Peña | Olga |
| Bejarano Redondo | Guillermina |
| Iglesias Sigüenza | Alberto |
| Tung Chen | Yale |
| Maroun Eid | Charbel |
| Bravo Lizcano | Ruth |
| Silvestre Niño | Miguel |
| Perdomo García | Frank |
| Alonso González | Berta |
| Antón Huguet | Berta |
| Arenas Berenguer | Isabel |
| Cabré-Verdiell Surribas | Clara |
| Marqués González | Francisco |
| Muñoz Del Val | Elena |
| Molina | María Ángeles |
| Cancelliere Fernández | Nataly |
| Pastor Yvorra | Sivia |
| Frade Pardo | Laura |
| López Arévalo | Paloma |
| García | Isabel |
| Internal Medicine | |
| Fernández Capitán | Carmen |
| González Garcia | Juan José |
| Herrero | Juan |

| | |
|---|---|
| Quesada Simón | María Angustias |
| Robles Marhuenda | Angel |
| Soto Abanedes | Clara |
| Noblejas Mozo | Ana María |
| Ramos | Juan Carlos |
| Jaras Hernandez | Maria Jesús |
| Martinez Robles | Elena |
| Moreno Fernandez | Alberto |
| Sanchez Purificación | Aquilino |
| Martin Gutiérrez | Juan Carlos |
| Martinez Hernández | Pedro Luis |
| Sancho Bueso | Teresa |
| Lorenzo Hernández | Alicia |
| Gutierrez Sancerni | Belén |
| Salgueiro | Giorgina |
| Martin Carbonero | Luz |
| Mostaza | Jose mAría |
| Martinez-López | María Angeles |
| Hontañon | Victor |
| Menéndez | Araceli |
| Alvarez Troncoso | Jorge |
| Castellano | Arancha |
| Marcelo Calvo | Cristina |
| Vives Beltrán | Ivo |
| Ramos Ruperto | Luis |
| Daroca Bengoa | German |
| Arcos Rueda | María |
| Vasquez Manau | Julia |
| Fernández Cidón | Pelayo |
| Herrero Gil | Carmen Rosario |
| Palmier Peláez | Esmeralda |
| Untoria Tabares | Yeray |
| Lahoz | Carlos |
| Estirado | Eva |
| Hernández | Clara |
| Garcia-Iglesias | Francisca |
| Monteoliva | Enrique |
| Martínez | Mónica |
| Varas | Marta |
| González Alegre | Teresa |
| Valencia | Maria Eulalia |
| Moreno | Victoria |
| Montes. | Maria Luisa |
| Pneumology | |
| Alcolea Batres | Sergio |
| Cabanillas Martín | Juan José |
| Carpio Segura | Carlos |
| Casitas Mateo | Raquel |
| Fernández-Bujarrabal Villoslada | Jaime |
| Fernández Navarro | Isabel |
| Fernández Lahera | Juan |

| | |
|---|---|
| García Quero | Cristina |
| Hidalgo Sánchez | María |
| Galera Martínez | Raúl |
| García Río | Francisco |
| Gómez Carrera | Luis |
| Gómez Mendieta | María Antonia |
| Mangas Moro | Alberto |
| Martínez Cerón | Elisabet |
| Martínez Redondo | María |
| Martínez Abad | Yolanda |
| Martínez-Verdasco | Antonio |
| Plaza Moreno | Cristina |
| Quirós Fernández | Sarai |
| Romera Cano | Delia |
| Romero Ribate | David |
| Sánchez Sánchez | Begoña |
| Santiago Recuerda | Ana |
| Villasante Fernández-Montes | Carlos |
| Zamarrón De Lucas | Ester |
| Arnalich Montiel | Victoria |
| Mariscal Aguilar | Pablo |
| Falcone | Adalgisa |
| Laorden Escudero | Daniel |
| Prados Sánchez | María Concepción |
| Álvarez-Sala Walther | Rodolfo |
| Intensive Care | |
| García | Andony |
| Arévalo | Cristina |
| Gutiérrez | Carola |
| Yus | Santiago |
| Asensio | Maria José |
| Sánchez | Manolo |
| Manuel Añón | Jose |
| Manzanares | Jesús |
| García De Lorenzo | Abelardo |
| Perales | Eva |
| Civantos | Belén |
| Cachafeiro | Lucía |
| Agrifoglio | Alexander |
| Estébanez | Belén |
| Flores | Eva |
| Hernández | Mónica |
| Millán | Pablo |
| Rodríguez | Montserrat |
| Nanwani | Kapil |
| Intensive Pediatry | |
| Arizcun | Beatriz |
| Pérez-Costa | Elena |
| Rodríguez-Álvarez | Diego |
| Sánchez-Martín | María |
| Quesada | Úrsula |

| | |
|---|---|
| Román-Hernández | Carmen |
| Dorao | Paloma |
| Álvarez-Rojas | Elena |
| Menéndez | Juan José |
| Verdú | Cristina |
| Gómez-Zamora | Ana |
| Schüffelmann | Cristina |
| Calderón-Llopis | Belén |
| Laplaza-González | María |
| Río-García | Miguel |
| Amores-Hernández | Irene |
| Rodríguez-Rubio | Miguel |
| de la Oliva | Pedro |
| Cardiology | |
| Ruiz | Jose |
| Rosillo | Sandra |
| González | Oscar |
| Iniesta | Angel |
| Ponz. | Ines |
| Anesthesiology | |
| Muñoz Ramón | José María |
| Hernández Gancedo | María Carmen |
| Uña Orejón | Rafael |
| Sanabria Carretero | Pascual |
| Moreno Gómez-Limón | Isidro |
| Seiz-Martinez | Alverio |
| Guasch-Arévalo | Emilia |
| Martín-Carrasco | Cristina |
| Alvar | Elena |
| Serrá | Lucía |
| Iannuccelli | Fabricio |
| Latorre | Julieta |
| Casares | Sandra |
| Valbuena | Isabel |
| Díaz Díez Picazo | Luis |
| Rodríguez Roca | Cristina |
| Cervera | Omar |
| García de las Heras | Esteban |
| Durán | Pilar |
| Castro | Carmen |
| Manrique de Lara | Carlos |
| Veganzones | Javier |
| López-Tofiño | Araceli |
| Fernandez-Cerezo | Estefanía |
| Zurita | Sergio |
| López-Martinez | Mercedes |
| Prim | Teresa |
| Alvárez Del Vayo | Julía |
| Alcaraz | Gabriela |
| Castro | Luis |
| Yagu¨e | Julio |

| | |
|---|---|
| Díaz-Carrasco | Sofía |
| González-Pizarro | Patricio |
| Montero | Ana |
| Sagra | Francisco Javier |
| Suárez. | Alejandro |
| Paliative Care | |
| Díez Porres | Leyre |
| Varela Cerdeira | María |
| Alonso Babarro | Alberto |
| Data Entry | |
| Abellán Martínez | Francisco |
| Alonso Eiras | Jorge Ignacio |
| Álvarez Brandt | Alejandra |
| Archinà | Martina |
| Arribas Terradillos | Silvia |
| Baselga Puente | Trinidad |
| Barco Núñez | Pilar |
| Barrera López | Natalia Guadalupe |
| Barrera López | Lorena |
| Bartrina Tarrio | Andres |
| Bassani | Gemma |
| Betancort De la Torre | Paula |
| Blanco Bartolomé | Irene |
| Blasco Andres | Celia |
| Brieba Plata | Lucia |
| Cadenas Gota | Fernando |
| Carrera Vázquez | Paloma |
| Cascajares Sanz | Carlota |
| Catino | Arianna |
| Cavallé Pulla | Raquel |
| Ceniza Pena | Daniel |
| Conde Alonso | Ylenia María |
| Currás Sánchez | Laura |
| Daltro Lage | Marcelo |
| Esteban Romero | Ana |
| Fernández Vidal | María Luisa |
| Ferrer Ortiz | Inés |
| de la Fuente Regaño | Lydia |
| Galindo Ballesteros | Pablo |
| Garcia-Bellido Ruiz | Sara |
| García-Mochales Fortún | Carlos |
| Gómez Ballesteros | Teresa |
| Gómez Domínguez | Cecilia |
| González Aguado | Nelsa |
| González García | Sofía |
| Guisández Martín | Jorge |
| Hernández Liebo | Paula Alejandra |
| Hernando Nieto | Raquel |
| Llorente Cortijo | Irene María |
| Marín García | Antonio |
| López Pirez | Pilar |

| | |
|---|---|
| Mejuto Illade | Lucía |
| Palma | Marco |
| Peña Hidalgo | Adrian |
| Platero Dueñas | Lucía |
| Pujol Pocull | David |
| Ramírez Verdyguer | Miguel |
| Redondo Gutierrez | Marta |
| Reinoso Lozano | Francisco |
| Rodríguez Revillas | Ana |
| Rodríguez Saenz de Urturi | Alejandro |
| Romero Imaz | Lucía |
| Sánchez Rico | Susana |
| Sánchez Santiuste | Mónica |
| Serrano de la Fuente | Patricia |
| Serrano Martín | Henar |
| Silva Freire | Thamires |
| Soria Alcaide | Eva |
| Suárez Plaza | Andrés Enrique |
| Tejero Soriano | Beatriz |
| Torrecillas Mainez | Andrea |
| Torres Cortés | Javier |
| Valentín-Pastrana Aguilar | María de Las Mercedes |
| Villanueva Freije | Angélica |
| Virgós Varela | Marta |
| Yagüe Barrado | Marta |
| Yustas Benitez. | Natalia |
| Risk Prevention | |
| Núñez | Mª Concepción |
| Clinical Pharmacology | |
| Montserrat | Jaime |
| Queiruga | Javier |
| Rodriguez Mariblanca | Amelia |
| Martínez de Soto | Lucía |
| Urroz | Mikel |
| Seco | Enrique |
| Zubimendi | Mónica |
| Stuart | Stephan |
| Díaz | Lucía |
| García. | Irene |
| management: | **Data** |
| García Morales | María Teresa |
| Martín-Vega | Alberto |
| Revision of data | |
| Caro | Abel |
| Martínez-Alés | Gonzalo |

**Table S2.** POSTCOVID@HULP Working Group.

| Department | Surname | Name |
|---|---|---|
| Medicine | Arnalich Fernández | Francisco |
| | Fernández Capitán | Carmen |
| | Salgueiro Origlia | Giorgina |

| | | |
|---|---|---|
| | Moreno Fernández | Alberto |
| Laboratory | Buño Soto | Antonio |
| | Qasem Moreno | Ana Laila |
| | Prieto Arribas | Daniel |
| Respiratory Medicine | Álvarez-Sala Walther | Rodolfo |
| | Gómez Carrera | Luis |
| | Carpio Segura | Carlos |
| | Mariscal Aguilar | Pablo |
| | Laorden Escudero | Daniel |
| | Arnalich Montiel | Victoria |
| Central Clinical Research Unit | Borobia Pérez | Alberto |
| | Jiménez González | María |
| | Rey Mauleón | Vega |
| Nursing | Alegre Segura | Carmen |
| | Cuesta Luzzy | Tania |
| | Martínez Gómez | Alejandra |
| | Moreno Juan | Ana María |
| | Rey Iborra | Cristina |
| | Sanz Jiménez | Andrea |
